# Perfectionism, test anxiety, and neuroticism determines high academic performance: a cross-sectional study

**DOI:** 10.1186/s40359-023-01369-y

**Published:** 2023-11-23

**Authors:** Jiyoon Shin, Hyung Jun Lee, Hyungyou Park, Yoontae Hong, Yong Keun Song, Dong Uk Yoon, Sanghoon Oh

**Affiliations:** 1 Department of Psychiatry, Seoul National University Hospital, Seoul, Republic of Korea; 2 YD Clinic Research Institute, Busan, Republic of Korea; 3 Department of Brain and Cognitive Sciences, Seoul National University College of Natural Sciences, Seoul, Republic of Korea; 4 Seoul National University College of Medicine, Seoul, Republic of Korea; 5 Department of Psychiatry, Uijeongbu Eulji Medical Center, Eulji University School of Medicine, Uijeongbu, Republic of Korea; 6 Department of Neurosurgery, Seoul National University Hospital, Seoul, Republic of Korea

**Keywords:** Academic high achievers, Psychological factors, Neuroticism, Test anxiety, Perfectionism

## Abstract

**Background:**

Academic performance is an important issue for Korean students. Various psychological factors contribute to academic performance. We aimed to evaluate the psychological factors that affect academic performance integratively.

**Methods:**

A total of 102 academic high achievers and 120 comparison participants were recruited. We evaluated psychological factors (test anxiety, perfectionism, personality traits, resilience, and self-efficacy) and measured academic performance using the College Scholastic Ability Test and the current college grade. We compared psychological factors and academic performance between the academic high achiever and comparison groups. Multiple linear regression was then conducted to identify the significant psychological factors for high academic performance. Further, we used cluster analysis to classify the comparison group by the significant psychological factors and compared them among clusters and academic high achievers to determine the psychological characteristics of academic high achievers.

**Results:**

The academic high achiever group showed lower test anxiety (*p* = .002), less neuroticism (*p* = .001), higher self-efficacy (*p* = .028), and less socially prescribed perfectionism (*p* < .001) than the comparison group. Multiple linear regression results (*p* = .020) clarified that neuroticism (*p = .*020), test anxiety level (*p* = .047), and perfectionism (*p* = .035) were important factors predicting better academic performance. Academic high achievers had moderate test anxiety and perfectionism levels, with the best performance on the College Scholastic Ability Test.

**Conclusions:**

Neuroticism, test anxiety levels, and perfectionism are important psychological factors for high academic performance. Interventions targeting these factors may help to improve academic accomplishments.

## Background

Academic performance is a major concern for South Korean high school students. This is because Korea’s social climate recognizes high academic performance in high school as a pathway to enter a top-ranked university, medical school or law school as a sign of social success in South Korea [1, 2]. As a result, high school students compete fiercely for admission to prestigious universities. This pressure to perform well on the College Scholastic Ability Test (CSAT) and other exams has led to a phenomenon known as the “university entrance exam hell.” In this social atmosphere, students experience chronic academic stress, leading to academic burnout, which results in psychological difficulties such as exhaustion, inefficacy, and poor academic performance [1, 3].

Test anxiety is one of the most important factors that cause academic burnout [4]. It refers to the emotional response and physiological changes that an individual experiences in a perceived situation [5]. The factors related to anxiety are stress from exams, worry about failure, lack of ability to respond appropriately to one’s situation, and tendency to react sensitively to others’ judgment [4, 6, 7]. In previous studies, the negative correlation between the test anxiety and academic performance is well replicated in other countries [6, 8, 9]. Those with high test anxiety were found to have a high need for approval and a perfectionist tendency to avoid tasks than to make a challenge [10]. People with socially prescribed perfectionism care excessively for other people’s gaze and are prone to depression, which also results in excessive test anxiety [10]. In a meta-analysis, perfectionistic concern is negatively correlated with academic performance, whereas perfectionistic striving is positively correlated with academic performance [11]. In addition, among the five-factor personality traits, conscientiousness and openness were found to have negative correlations with test anxiety, whereas neuroticism was positively correlated [12]. Neuroticism, composed of negative psychological subscales such as anxiety, anger, and depression, is related to lower academic performance and is the factor that best explains test anxiety [13].

Previous studies have identified additional psychological characteristics necessary for good academic performance. Resilience acts as a buffer against test anxiety and academic burnout. Resilience is the ability to significantly reduce stress and to be less affected by stressful situations [4]. Subscales such as self-regulating ability, interpersonal ability, and positivity constitute resilience [14]. Individuals with high resilience have the ability to overcome difficulties, turning them into successful experiences, resulting in outstanding academic performance [15, 16]. Resilience interventions help improve stress-coping strategies in college students during increased academic stress [17]. The stress-coping method is another crucial factor for high academic performance [18]. When using active stress-coping methods rather than passive methods, students can effectively cope with academic stress [1]. Active coping has been shown to reduce academic burnout by suppressing intrinsic problems such as depression and anxiety [19].

Self-efficacy is an important factor related to academic resilience [20]. Self-efficacy had a positive effect on school adaptation and academic performance. Self-efficacy is the belief that one can organize the actions required to perform a task [21]. It appears to affect academic performance by influencing decision-making, persistence, perseverance, and effort in necessary behaviors [21, 22].

The study aims to comprehensively evaluate the psychological factors affecting academic performance in medical students with high academic performance. The study will compare the academic high achiever (AHA) group to a comparison group of students who are pursuing majors other than medicine in a college located in Seoul. The study hypothesizes that the AHA group will exhibit better stress coping strategies, lower levels of neuroticism, test anxiety, and perfectionism, higher levels of resilience, and higher self-efficacy compared to the comparison group. The study also hypothesizes that these psychological factors will correlate with academic performance.

## Methods

### Participants

The study included 102 participants in the AHA group, who were students from the medical school of Seoul National University (1st and 2nd -grade premedical students and medical students from 1st to 4th grade), and 120 comparison participants from universities located in Seoul (1st to 4th grade) excluding medical schools. In South Korea, medical school enrollment is allowed only in the top 0.1% ranking of academic scores. Among them, the College of Medicine at Seoul National University is the top-ranked school. Students from the College of Medicine, Seoul National University, were recruited with consent from the Student Counsel of the university with a recruitment notice on ‘Kakao talk,’ a group chat room widely used in Korea. The comparison participants were recruited using online university bulletin boards. We did not include other medical school students in the comparison group because the CSAT scores of other medical school students are also at the top tier in South Korea. We posted a research recruitment notice on the app. The link for the survey was provided by KakaoTalk and online bulletin boards. The participants were recruited between November 2020 and January 2021. The study was approved by the Institutional Review Board for Human Subjects of the Pusan National University Yangsan Hospital (IRB no. 05-2020-195). Informed consent was obtained from all participants before entering the study.

### Assessments

All participants were assessed by online self-report questionnaires using the Ways of Coping Checklist (WCC), Big Five Inventory-Korean Version (BFI-K), Revised Test Anxiety Scale (RTA), Korean Resilience Questionnaire (KRQ), Multi-dimensional Perfectionism Scale (MPS), and Academic Self-Efficacy (ASE). Participants reported their past and current academic performance.

### Ways of coping checklist

The WCC is a scale that determines how an individual copes with stress and is classified into active and passive coping strategies [23, 24]. Subscales comprised ‘problem-focused coping,’ ‘seeking social support,’ ‘emotion-focused coping,’ and ‘wishful thinking.’ The first two subscales are classified into active coping strategies and the latter two into passive coping strategies. Active coping means that the target of the effort is directed outward, whereas the effort of passive coping is directed inward, such as one’s feelings or thoughts. Each item is on a 4-point scale and consists of 64 items. In a study by Kim et al., Cronbach’s alpha was 0.67 to 0.95 [24]. In our study, the value ranged from 0.68 to 0.87.

### Big five inventory-Korean version

A total of 44 items in the BFI-K measure different aspects of personality traits (extraversion, agreeableness, conscientiousness, neuroticism, and openness), which measure the degree of sociality, maintaining harmonious relationships with others, tendency to adhere to social norms, emotional stability, and pursuit of intellectual stimuli, respectively [25]. It is scored on a 5-point Likert scale, and the average value is obtained by summing the original score. A higher score indicates a stronger personality tendency. In a study of BFI-K, internal consistency was good, and Cronbach’s alpha ranged from 0.89 to 0.94 [26]. In this study, Cronbach’s alpha value of extraversion was 0.61, and other subscales of the BFI-K ranged from 0.73 to 0.82.

### The revised test anxiety scale

This scale was developed to measure test anxiety [27]. It consists of 20 items with 4 factors:5 items for tension, 6 for worry, 5 for bodily symptoms, and 4 for test-irrelevant thinking. The tension subscale consists of items on tension, anxiety, and uneasy feelings related to the test. The worry subscale consists of thinking about the test results and comparing them with others. The bodily symptom subscale assesses physical discomfort. Items such as “I think about current events during a test” and “During a test, I find I am distracted by thoughts of upcoming events” constitute the test-irrelevant thinking subscale [27]. The scale is classified into three levels: low (≤ 37 scores), moderate (38–50 scores), and high anxiety (≥ 51 scores). The overall reliability of the scale was relatively high in the original study (Cronbach’s alpha = 0.89) [27]. This study had a Cronbach’s alpha of 0.91.

### Korean resilience questionnaire

The KRQ consists of 27 items, each evaluated on a 5-point scale [28, 29]. Resilience is defined as a multi-dimensional ability composed of 9 dimensions: causal analysis, emotion control, impulse control, gratitude, life satisfaction, optimism, relationship, communication ability, and empathy. The total score ranged from 27 to 135, with higher scores indicating higher resilience. In a validation study of the KRQ, Cronbach’s alpha was 0.67–0.85 in college students [29]. Our study showed low internal consistency in causal analysis (Cronbach’s alpha = 0.66), emotion control (Cronbach’s alpha = 0.60), and impulse control (Cronbach’s alpha = 0.33). The other subscales showed good internal consistency ranging from 0.68 to 0.88.

### Multi-dimensional perfectionism scale

MPS regards perfectionism as a multi-dimensional construct that includes personal and social aspects [30]. It is composed of 45 items on a 7-point scale, and the scale is composed of three subscales: self-oriented, other-oriented, and socially prescribed perfectionism. Self-oriented perfectionism refers to setting high standards and rigorously evaluating one’s behavior to achieve perfection. Other-oriented perfectionism is the tendency to judge others strictly by setting high standards for them and criticizing and distrusting them. Socially prescribed perfectionism is related to the belief that an important person sets unrealistic standards and expects them to reach the objective. It is known that the higher the score, the higher the tendency toward perfection. In Hewitt and Flett’s study, Cronbach’s alpha ranged from 0.74 to 0.88 [30, 31], and in our study, the values were 0.73 to 0.89. In a validation study of the Korean version of the MPS, the reliability coefficient of the total scale was 0.86 [32].

### Academic self-efficacy (ASE)

The self-efficacy scale measures people’s confidence in their abilities. The academic self-efficacy scale consists of three categories: confidence (8 items), self-regulation efficacy (10 items), and task-level preference (10 items) [33]. Confidence refers to the students’ degree of confidence in their ability to learn. Self-regulation efficacy measures the expectations of self-observation, self-judgment, and self-response related to learning. Task-level preferences evaluate the tendency to choose challenging goals. The items were evaluated on a 6-point Likert scale. In the validation study of the ASE, Cronbach’s alpha was 0.74–0.84 [33]. Our study showed good internal consistency (Cronbach’s alpha 0.83–0.90).

### Academic performances

Academic performance was evaluated in 2-dimensions, past and current. The past performance was measured using CSAT. The examination included Korean, mathematics, English, science, and social sciences. Each subject was graded from 1 (highest level, top 4 percentile) to 9 (lowest level). The total CSAT was calculated by summing the scores of Korean, mathematics, and English. We excluded science and social science scores from the total CSAT scores because students had various sub-subjects to choose from.

For current academic performance, students were asked about their perceived academic performance in the school by the questionnaire “What is the current average grades in college?”. It was scored on a 5-point Likert scale, ranging from 1 (very poor) to 5 (very good).

### Statistical analyses

Continuous and categorical demographic data were analyzed using Student’s t-test and Chi-square tests, respectively, to compare AHA with comparison group. To compare the group-ratio differences between the two groups, test anxiety was categorized into three groups: high, moderate, and low anxiety levels. Based on a previous paper [6, 34], we calculated the scores by categorizing them into low, moderate, and high anxiety levels, to provide meaningful clinical implications.

To adjust for differences in age and gender between the two groups, we conducted an additional analysis of covariance. We adjusted for gender in the first model and both age and gender in the second model. To examine the essential psychological factors for academic performance, multiple linear regression was conducted using the past academic performance of comparison group as a dependent variable and psychological factors as independent variables. Psychological factors that showed significant differences between the AHA and comparison groups were selected as independent variables. We used the backward method to evaluate the multiple linear regression model with the significant factors. Further subgroup analysis was conducted by stratifying the data by gender. The model revealed by the multiple linear regression above was applied to each gender group separately.

In addition, we performed a cluster analysis to classify the comparison groups by the significant psychological factors (standardized Z-score) revealed by multiple linear regression. We performed a two-phase cluster analysis. First, we performed a hierarchical cluster analysis using Ward’s agglomerative method to classify the comparison group. The number of clusters was determined using the dendrogram, agglomeration coefficients, and interpretability of the classification [35]. Next, we used K-means cluster analysis to confirm the number of groups identified by hierarchical clustering. Academic performance was then compared among clusters identified by one-way analysis of variance. We conducted a post-hoc analysis to compare total CSAT scores among the groups. The Bonferroni method was used for variables with homogeneous variances, and the Dunnett T3 method was used for variables with heterogeneous variances.

All analyses were performed using SPSS version 25.

## Results

### Demographics and performance characteristics

The AHA group (n = 102) was significantly older than the comparison students (n = 120). The AHA group comprised more men than the comparison group. Performances in the CSAT scores of the AHA were better in all subjects: Korean, mathematics, English, science, and social studies (Table 1). The age, academic year, and CSAT level of each subject presented heterogeneous variances.

**Table 1:** Demographics and CSAT scores of participants

	AHA	Comparison	t/χ2	Effect size	p-value
	(n = 102)	(n = 120)			
Age	22.8 ± 3.0	21.2 ± 2.1	t = 4.77	d = 0.63	< 0.001
Gender (male/female)	59/43	44/76	χ^2^ = 9.94	φ = 0.21	0.002
Academic year	3.7 ± 1.6	2.3 ± 1.2	t = 7.24	d = 0.97	< 0.001
CSAT level					
Korean	1.2 ± 0.5	2.4 ± 1.3	t=-8.56	d = 1.30	< 0.001
Mathematics	1.2 ± 0.5	2.4 ± 1.4	t=-8.04	d = 1.22	< 0.001
English	1.1 ± 0.6	2.1 ± 1.0	t=-7.84	d = 1.15	< 0.001
Science or social studies (1)	1.3 ± 0.6	2.2 ± 1.2	t=-6.52	d = 0.98	< 0.001
Science or social studies (2)	1.5 ± 0.7	2.4 ± 1.3	t=-6.26	d = 0.94	< 0.001
Current academic performance	3.3 ± 0.9	3.6 ± 0.8	t=-2.74	d = 0.38	0.007

AHA: Academic high achiever. CSAT: College Scholastic Ability Test (Level 1 (highest performance) to Level 9 (worst performance)). Current academic performance (1 (lowest performance) to 5 (highest performance)). d: cohen’s d. φ: phi coefficient

### Psychological factor differences between AHA vs. comparison

**Fig. 1:**
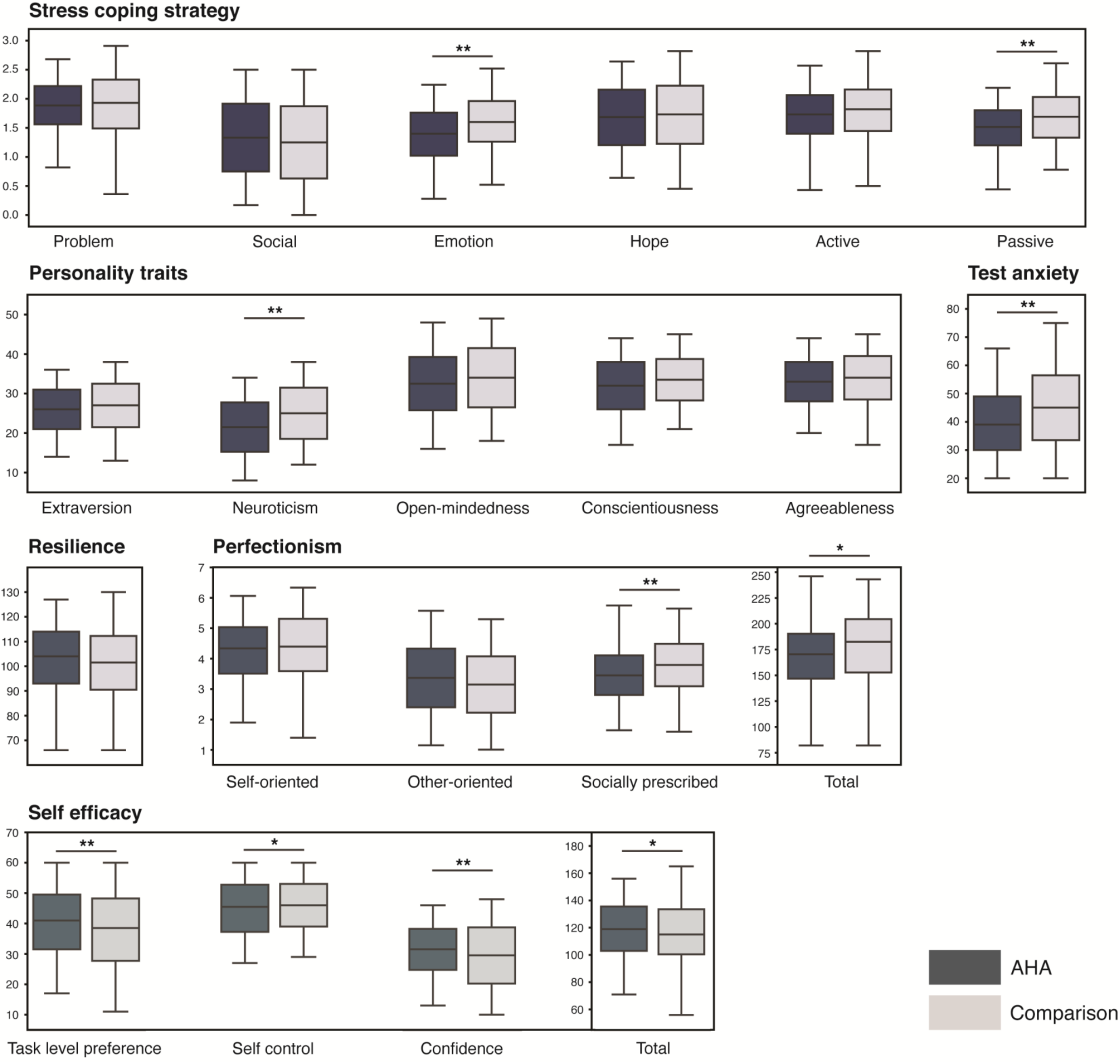
Group differences in psychological scores between academic high achievers (AHA, n = 102) and comparison group (n = 120). The results from the student t-test are presented as “*” (p < .05) and “**” (p < .01) on the graph

We analyzed the differences in psychological factors between the AHA and comparison groups (Fig. 1). The total test anxiety and confidence subscale in self-efficacy presented inhomogeneous variance, while other psychological variables showed homogeneous variances.

Among the stress coping strategies, AHA used less emotion-focused coping than the comparison group (*t*=-4.65, *p* < .001). The passive coping strategy score, the sum of wishful thinking and emotion-focused coping subscales, was also lower in AHA than in the comparison group (*t*=-4.05, *p* < .001). However, the two groups had no differences in the frequency of active coping strategy use.

The neuroticism personality scores were lower in AHA (*t*=-3.49, *p* = .001). There were no differences in the other four personality scales.

Regarding test anxiety, the total anxiety score was significantly higher in the comparison group. The ratio of high- to low-level anxiety students was also higher among comparison group (*χ2* = 10.45, *p* = .001). To be specific, 12 AHA participants comprised high level anxiety, 41 in moderate level anxiety, and 49 in low level anxiety group, whereas, the number was 35, 44, and 41 in comparison group.

Socially prescribed perfectionism was lower in AHA (*t*=-3.63, *p* < .001). Regarding the academic self-efficacy scale, the AHA had higher task difficulty scores (*t* = 3.32, *p* = .001), meaning they preferred to challenge tasks and had lower self-confidence.

The psychological factors showed significant differences between the AHA and comparison groups after adjusting for gender (all p-values < 0.05). The differences remained significant after adjusting for both gender and age (all p-values < 0.01).

Total KRQ score and three subscales of the scale did not show differences between the two groups.

### Factors Predicting higher academic performance

**Fig. 2:**
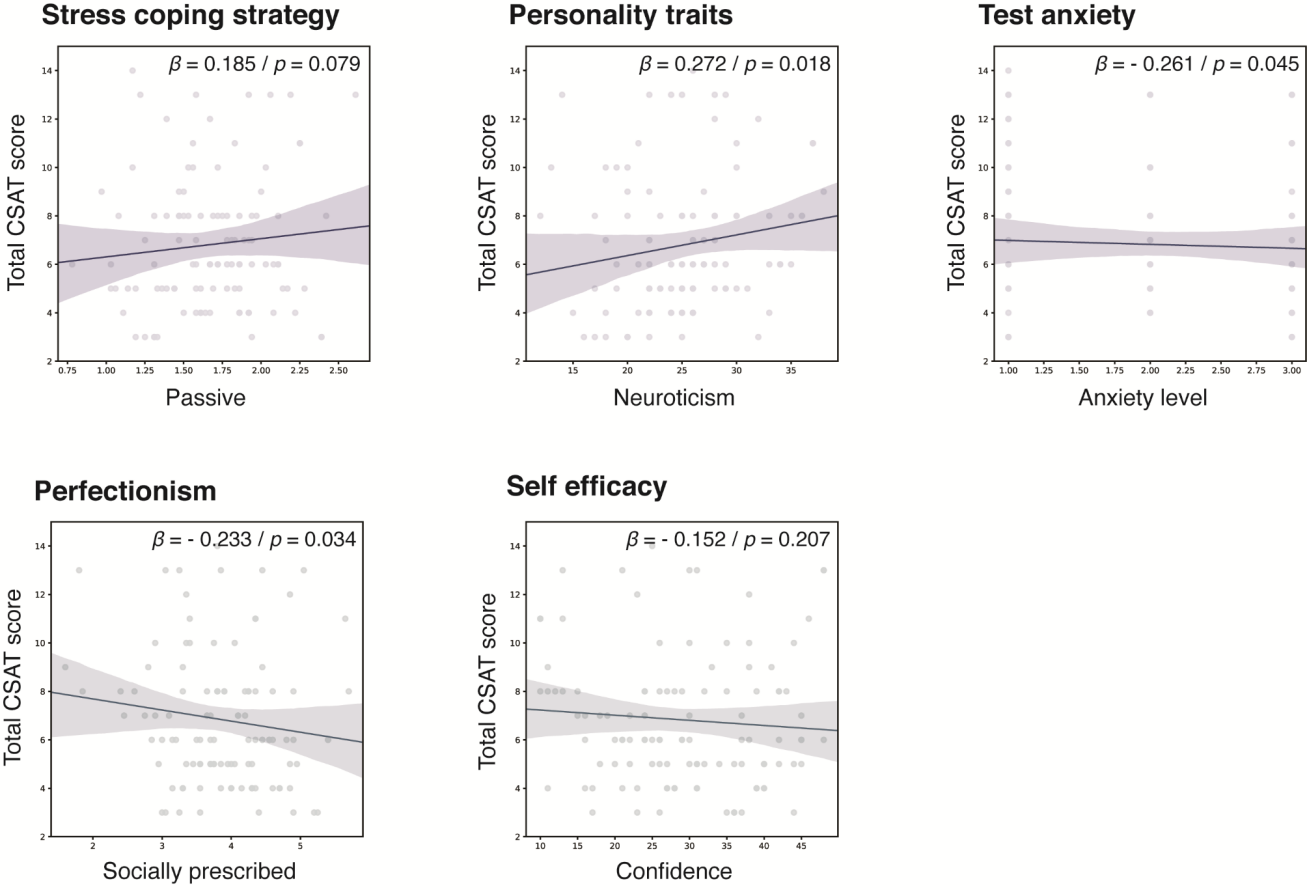
Multivariate linear regression analysis for the prediction of academic performance in the comparison group. Graphs present the relationship between psychological factors and the total score of the College Scholastic Ability Test (CSAT), where a lower score on the CSAT indicates a higher grade. CSAT: College Scholastic Ability Test

As independent variables, psychological factors showing differences between the AHA and comparison groups (Fig. 2) were included in multiple linear regression analysis, which were passive coping strategy, neuroticism, test anxiety level, socially prescribed perfectionism, task level preference, and confidence in self-efficacy. The model was significant in the comparison group (*p* = .020), and the factors predicting better performance in total CSAT scores were neuroticism, anxiety level, and socially prescribed perfectionism (Fig. 2). Task level preference and self-control were eliminated in the model using backward method. Specifically, higher academic performance is predicted by lower neuroticism, higher anxiety levels, and higher socially prescribed perfectionism. Other independent variables included in the model were passive stress coping strategy, task-level preference, and confidence, which were found to be insignificant. The predictive model derived from comparison group was not significant for AHA (p = .540) and no significant model was calculated with multiple linear regression using backward method.

The subgroup analyses with gender stratification in the comparison group utilized the same predictive model derived from the whole comparison group. Models for both males and females were found to be insignificant (p = .107 for males and p = .622 for females).

### Cluster analysis and academic performance comparison among clusters

The hierarchical analysis results suggest a three-cluster solution classified by neuroticism, test anxiety, and perfectionism. The K-means cluster specified solutions with significant differences in neuroticism, total test anxiety, and total perfectionism scores (Table 2). Cluster 1 (perfectionists with test anxiety) had high levels of all three characteristics, whereas cluster 3 (no perfectionism and test anxiety) had low levels of all three characteristics. Analysis of variance revealed that academic performances in Korean, English, and total CSAT were inferior in Cluster 3 compared to Cluster 2 (perfectionists without test anxiety). Furthermore, AHA had lower perfectionism than Cluster 2 and presented the best academic performance.

**Table 2:** Descriptive statistics and comparison of psychological factors and academic performances

	Cluster 1			Cluster 2			Cluster 3			AHA		df	F	p-value	Partial η2	Post-hoc analysis	
	n = 38			n = 52			n = 22			n = 101							
	M	SD	Z	M	SD	Z	M	SD	Z	M	SD						
Neuroticism	29.95	4.40	2.48	23.48	3.71	-0.86	20.78	5.11	-2.09	22.48	5.65	3, 217	25.52	< 0.001	0.26	C1 > C2 = C3 = A	
Test anxiety	58.28	7.83	2.34	38.07	7.25	-1.21	32.26	7.23	-1.72	38.65	9.97	3, 217	63.65	< 0.001	0.47	C1 > C2 = A > C3	
Perfectionism	193.92	19.65	0.45	191.47	19.88	1.81	132.87	23.17	-3.38	170.03	29.16	3, 217	25.84	< 0.001	0.35	C1 = C2 > A > C3	
	n = 39			n = 58			n = 23			n = 101							
Korean	2.26	1.11		2.19	1.25		3.09	1.28		1.15	0.45	3, 192	30.23	< 0.001	0.32	A > C1 = C2 > C3	
Math	2.53	1.33		2.23	1.23		2.77	1.66		1.20	0.46	3, 192	22.95	< 0.001	0.26	A > C1 = C2 = C3	
English	2.21	0.99		1.79	0.91		2.43	0.99		1.11	0.60	3, 192	24.82	< 0.001	0.28	A > C2 > C3, A > C1	
Total CSAT	7.00	2.56		6.20	2.68		8.05	2.92		3.46	1.06	3, 192	43.36	< 0.001	0.41	A > C2 > C3, A > C1	
Current grade	3.56	0.88		3.74	0.91		3.62	0.89		3.31	0.89	3, 192	3.13	0.026	0.042	A > C1 = C2 = C3	

M: mean. SD: standard deviation. Z: standardized Z-score. C1: Cluster 1, C2: Cluster 2, C3: Cluster 3, A: AHA. Total CSAT: sum of Korean, Math, and English scores

## Discussion

Our study is the first to compare one of the highest academic performance groups in South Korea with the comparison group to investigate the important psychological factors for excellent academic performance. We found that neuroticism, test anxiety levels, and perfectionism were important psychological factors related to high academic performance.

Several psychological characteristics of AHA showed differences compared to the comparison group, which may be critical psychological characteristics of students with high academic performance.

AHA had lower neuroticism and emotion-focused coping scores, which seems to be interrelated. Neuroticism traits tend to include emotional instability, characterized as being anxious, emotional, nervous, and jealous [36, 37]. Using less emotion-focused coping means they made less effort to control their stress-induced emotions. Taken together, it can be interpreted that less neurotic AHA needed less effort to regulate their emotions than did the comparison group. Emotional stability, meaning less neuroticism [36], can help students concentrate on their studies and achieve better academic performance. Furthermore, our analyses showed that higher neuroticism predicted lower grades, consistent with previous studies suggesting a dysfunctional role in performance.

AHA had higher academic self-efficacy than the comparison group. Self-efficacy is the belief that one can perform a specific task and organize the necessary behavior to complete the task successfully [21]. It also affects the ability to persist, persevere, and strive for action [21]. Past successful experiences, such as high CSAT scores, seem to have contributed to this increased sense of self-efficacy. However, the AHA group had lower self-control efficacy than the comparison group. Since self-regulation is one’s capacity to plan, control, and adapt internal status to achieve goals in demanding environments [38], the result might reflect that AHA have difficulties in emotional adaptation to their academic grades in a highly competitive medical school environment.

Socially prescribed perfectionism and test anxiety scores of the AHA group were lower than those of the comparison group, consistent with previous studies [2, 6]. These results may explain why these lower characteristics are related to better academic performance. However, linear regression analyses of the comparison group revealed that the stronger the characteristics, the higher the academic performance. These results suggest that the relationships of academic performance with socially prescribed perfectionism and test anxiety scores are not simply linear. The inverted U-shaped relationship between test anxiety and academic performance, Yerkes-Dodson law [34], partially explains our non-linear results.

However, our linear regression model was not significant for AHA. This might be because the CSAT score is a categorical variable rather than a continuous one, divided by nine levels according to the percentage of the rank of a student. Most of participants in the AHA group received level 1, the highest level, in most of the subjects in the CSAT, so it was difficult to distinguish by grade within the group. Existing studies on the relationship between socially prescribed perfectionism and academic performance are scarce. Still, it can be assumed that this might also show a similar curve of an inverted U-shape as test anxiety [6, 34]. However, further studies are needed to clarify the relationship between socially prescribed perfectionism and academic performance.

To specify the results of the regression analysis, we conducted cluster analysis. Our cluster analysis in comparison students showed that cluster 3, which presented lower total perfectionism and test anxiety scores compared to cluster 2, scored the lowest in total CSAT. These results suggest that a healthy level of perfectionism and test anxiety may be essential for academic performance. AHA had moderate perfectionism and test anxiety, scoring the highest on the CSAT. Further, AHA had no differences in neuroticism and test anxiety but lower perfectionism than Cluster 2 (perfectionists without test anxiety). Taken together, these results imply that adaptive levels of test anxiety and perfectionism can help achieve high academic performance. Further studies are needed to determine the adaptive levels of test anxiety and perfectionism, which can be helpful intervention points for students.

We hypothesized that students with lower perfectionism would exhibit better academic performance. However, AHA presented a moderate level of perfectionism, with lower scores in socially prescribed perfectionism compared to comparisons, which might reflect low perfectionistic concerns [39, 40]. People with high perfectionistic striving and low perfectionistic concerns are regarded as “healthy perfectionists” [39]. People with high perfectionistic striving are motivated by the desire to achieve high standards of personal performance [40]. They strive for perfect performance and are often highly organized in the goal-achievement process [40]. In a previous study, positive striving perfectionism was an important factor in gifted achievers compared to underachievers [18]. Conversely, people with perfectionist concerns have the characteristics of self-blame, venting, and behavioral disengagement [41], which can lead to low academic performance. Taken together, healthy perfectionism may be a discriminating factor for superior academic performance.

Our study has several limitations. First, the CSAT is divided into nine levels according to rank percentage, which is not a continuous variable. Most participants in AHA received grade 1 in Korean, English, and mathematics, so there was a limit to elucidating the significant psychological factors among AHA. Second, the comparison group included college students from various schools, but they also showed high average scores on level two. High school students with good CSAT scores tended to apply to universities in the capital city of Seoul, which may have a selection bias. Recruitment was conducted only in Seoul, so it may be difficult to generalize the results to the general population. Further studies are needed, including students in other areas of Korea, to accurately predict the relationship between academic performance and psychological factors. Third, we only used self-report measures in the study, which may have caused recall bias. However, given the significance of the CSAT score to high school and college students, it is possible that their recall is much precise. Further studies with objective measure of academic performance are needed to confirm our results. Fourth, this was a cross-sectional study, which has limitations in eliciting cause and effect. Nevertheless, it is worth noting that perfectionism, test anxiety, and neuroticism can be considered relatively stable trait characteristics [42–44], that can influence academic performance. Therefore, further intervention studies targeting these factors could help validate the causal relationship between them and academic performance. Fifth, the AHA and comparison groups showed differences in gender ratio, which could impact the psychological characteristics of the groups. However, we conducted a comparison adjusting for gender, in which the results remained significant. Furthermore, additional subgroup analysis using gender stratification in predictive models was not significant in both gender groups that we could not conclude the gender effect on our findings. Therefore, further longitudinal studies with larger sample size are necessary to explain the relationship between the identified factors, considering the effect of gender.

## Conclusions

This study found that neuroticism, anxiety levels, and perfectionism are important factors in academic performance. With further analysis, the adaptive levels of test anxiety and perfectionism may play a crucial role in achieving high levels of achievement. Therefore, interventions such as cognitive-behavioral therapy, biofeedback, and relaxation therapy targeting these factors could help improve academic accomplishments.

## Data Availability

The datasets used and/or analysed during the current study available from the corresponding author on reasonable request.

## List of abbreviations

AHA: academic high achievers
ASE: Academic Self-Efficacy
BFI-K: Big Five Inventory-Korean Version
CSAT: College Scholastic Ability Test
KRQ: Korean Resilience Questionnaire
MPS: Multi-dimensional Perfectionism Scale
RTA: Revised Test Anxiety Scale
WCC: Ways of Coping Checklist

## References

[1] Shin H, Choi H, Lee M, Noh HK, Kim K, Jang Y (2012). The Effects of coping strategies on academic burnout: a short-term longitudinal study focused on suppression Effects. Korean J School Psychol.

[2] Lee SL, Jungyoon (2019). The Mediating Effects of difficulties in emotion regulation on the relationship between repeaters’ evaluative concern perfectionism, Personal Standard Perfectionism and Academic Burnout. Asian J Educ.

[3] Schaufeli WB, Martinez IM, Pinto AM, Salanova M, Bakker AB (2002). Burnout and engagement in university students: a cross-national study. J Cross Cult Psychol.

[4] Yune S-JI, Lee S-J, Baek S-Y, Kam S-Y (2018). Relationships among Test anxiety, academic burnout, resilience, and academic achievement of Medical School Students. J Educational Innov Res.

[5] Segool NK, Carlson JS, Goforth AN, Von Der Embse N, Barterian JA (2013). Heightended test anxiety among young children: Elementary school students’ anxious responses to high-stakes testing. Psychol Sch.

[6] Cassady JC, Johnson RE (2002). Cognitive test anxiety and academic performance. Contemp Educ Psychol.

[7] Chapell MS, Blanding ZB, Silverstein ME, Takahashi M, Newman B, Gubi A (2005). Test anxiety and academic performance in undergraduate and graduate students. J Educ Psychol.

[8] Safeer U, Shah SA (2019). Effect of test anxiety on academic achievement of university students. Pakistan J Physiol.

[9] Parveen S, Aijaz A, Rizvi SZS (2019). Tes anxiety and academic performance among senior secondary students. Indian J Appl Res.

[10] Deffenbacher JL, Zwemer WA, Whisman MA, Hill RA, Sloan RD (1986). Irrational beliefs and anxiety. Cognit Ther Res.

[11] Madigan DJ (2019). A meta-analysis of perfectionism and academic achievement. Educ Psychol Rev.

[12] Zhang L-f (2003). Does the big five predict learning approaches?. Pers Individ Dif.

[13] Kim A-R. The effect of big five personality factor and test-anxiety on the academic achievement. Dissertation of Graduate school of educational psychology, Sookmyung Women’s University. 2005.

[14] Luthar SS (1991). Vulnerability and resilience: a study of high-risk adolescents. Child Dev.

[15] Rutter M (1985). Resilience in the face of adversity: protective factors and resistance to psychiatric disorder. Br J Psychiatry.

[16] Polk LV (1997). Toward a middle-range theory of resilience. Adv Nurs Sci.

[17] Steinhardt M, Dolbier C (2008). Evaluation of a resilience intervention to enhance coping strategies and protective factors and decrease symptomatology. J Am Coll Health.

[18] Mofield E, Parker Peters M, Chakraborti-Ghosh S (2016). Perfectionism, coping, and underachievement in gifted adolescents: Avoidance vs. approach orientations. Educ Sci.

[19] Gaylord-Harden NK, Cunningham JA, Holmbeck GN, Grant KE (2010). Suppressor effects in coping research with african american adolescents from low-income communities. J Consult Clin Psychol.

[20] Cassidy S (2015). Resilience building in students: the role of academic self-efficacy. Front Psychol.

[21] Bandura A (1977). Self-efficacy: toward a unifying theory of behavioral change. Psychol Rev.

[22] Kim H-M. The Effect of Academic Self-Efficacy and Career Decision Level on Career Preparation Behavior of South Korean College Student. Dissertation of the graduate school, department of psychology, Handong Global University. 2019.

[23] Lazarus RS, Folkman S. Stress, appraisal, and coping. Springer publishing company; 1984.

[24] Kim YJ. The relationships among communication in parent-child, stress coping and adolescents’ school adjustment. Dissertation of Graduate school of Counseling Psychology, Seoul Women’s University. 2006.

[25] Pervin LA, John OP. Handbook of personality. Theory and research. 1999;2.

[26] Ha D, Hwang H, Nam S (2008). The development of big 5 personality inventory and its criterion-related validity on school grade, school adaptation, and career selection. Korean J Educ Psychology.

[27] Benson J, El-Zahhar N (1994). Further refinement and validation of the revised test anxiety scale. Struct Equation Modeling: Multidisciplinary J.

[28] Reivich K (2003). The resilience factor: 7 Keys to finding your Inner Strength and Overcoming Life’s hurdles.

[29] Shin W-Y, Kim M-G, Kim J-H (2009). Developing measures of resilience for korean adolescents and testing cross, convergent, and discriminant validity. Stud Korean Youth.

[30] Hewitt PL, Flett GL (1991). Perfectionism in the self and social contexts: conceptualization, assessment, and association with psychopathology. J Pers Soc Psychol.

[31] Frost R (1990). The dimensions of perfectionism. Cogn Ther Res.

[32] Hong HY. The Relationship of Perfectionism, Self-Efficacy and Depression. Dissertation of the graduate school, department of psychology, Ewha Womans University. 1994.

[33] Kim A, Park I-y (2001). Construction and valiation of academic self-efficacy scale. J Educational Res.

[34] Yerkes RM, Dodson JD. The relation of strength of stimulus to rapidity of habit-formation. Punishment: Issues and Experiments. 1908:27–41.

[35] Aldenderfer MS, Blashfield RK (1984). Cluster analysis.

[36] De Feyter T, Caers R, Vigna C, Berings D (2012). Unraveling the impact of the big five personality traits on academic performance: the moderating and mediating effects of self-efficacy and academic motivation. Learn Individual Differences.

[37] Noftle EE, Robins RW (2007). Personality predictors of academic outcomes: big five correlates of GPA and SAT scores. J Pers Soc Psychol.

[38] Zimmerman BJ. Attaining self-regulation: A social cognitive perspective. Handbook of self-regulation: Elsevier; 2000. p. 13–39.

[39] Gotwals JK, Spencer-Cavaliere N (2014). Intercollegiate perfectionistic athletes’ perspectives on achievement: contributions to the understanding and assessment of perfectionism in sport. Int J Sport Psychol.

[40] Holt NL (2014). A person-oriented examination of perfectionism and slump-related coping in female intercollegiate volleyball players. Perfectionism in Sport and Dance.

[41] Flett GL, Hewitt PL (2014). The perils of perfectionism in sports revisited: toward a broader understanding of the pressure to be perfect and its impact on athletes and dancers. Int J Sport Psychol.

[42] Stoeber J, Otto K (2006). Positive conceptions of perfectionism: approaches, evidence, challenges. Pers Soc Psychol Rev.

[43] Ormel J, Jeronimus BF, Kotov R, Riese H, Bos EH, Hankin B (2013). Neuroticism and common mental disorders: meaning and utility of a complex relationship. Clin Psychol Rev.

[44] Ping LT, Subramaniam K, Krishnaswamy S (2008). Test anxiety: state, trait and relationship with exam satisfaction. Malaysian J Med Sciences: MJMS.

